# Remoteness and distance, distance (signless) Laplacian eigenvalues of a graph

**DOI:** 10.1186/s13660-018-1663-5

**Published:** 2018-04-03

**Authors:** Huicai Jia, Hongye Song

**Affiliations:** 1 College of Science, Henan Institute of Engineering, Zhengzhou, P.R. China; 2 Department of Mathematics, School of Information, Renmin University of China, Beijing, P.R. China; 3 School of General Education, Beijing International Studies University, Beijing, P.R. China

**Keywords:** 05C50, Remoteness, Distance eigenvalues, Distance (signless) Laplacian eigenvalues

## Abstract

Let *G* be a connected graph of order *n*. The remoteness of *G*, denoted by *ρ*, is the maximum average distance from a vertex to all other vertices. Let $\partial_{1}\geq\cdots\geq\partial_{n}$, $\partial_{1}^{L}\geq\cdots\geq\partial_{n}^{L}$ and $\partial_{1} ^{Q}\geq\cdots\geq\partial_{n}^{Q}$ be the distance, distance Laplacian and distance signless Laplacian eigenvalues of *G*, respectively. In this paper, we give lower bounds on $\rho+\partial _{1}$, $\rho-\partial_{n}$, $\rho+\partial_{1}^{L}$, $\partial_{1} ^{L}-\rho$, $2\rho+\partial_{1}^{Q}$ and $\partial_{1}^{Q}-2\rho$ and the corresponding extremal graphs are also characterized.

## Introduction

In this paper, we consider simple, undirected and connected graphs. Let *G* be a graph with vertex set $V(G)$ and edge set $E(G)$, where $\vert V(G)\vert =n$, $\vert E(G)\vert =m$. Let *δ* be the minimum degree of the graph *G*. The distance between vertices $v_{i}$ and $v_{j}$ is the length of a shortest path connecting them in *G*, denoted by $d_{ij}$. The diameter of a graph is the maximum distance between any two vertices of *G*, denoted by *d*. The transmission $\operatorname{Tr}(v_{i})$ of vertex $v_{i}$ is defined to be the sum of distances from $v_{i}$ to all other vertices and *G* is transmission regular if $\operatorname{Tr}(v_{1})=\cdots=\operatorname{Tr}(v _{n})$. The *remoteness* *ρ* of *G* is denoted by

$$\rho=\rho(G)=\max_{v\in V(G)}\frac{\operatorname{Tr}(v)}{n-1}. $$

The distance matrix of *G*, denoted by $D(G)$, is the symmetric real matrix with $(i, j)$-entry being $d_{ij}$. Let $\operatorname{Tr}(G)=\operatorname{diag}(\operatorname{Tr}(v _{1}), \operatorname{Tr}(v_{2}), \ldots, \operatorname{Tr}(v_{n}))$ be the diagonal matrix of the vertex transmissions in *G*. The distance Laplacian matrix and the distance signless Laplacian matrix of *G* are defined as $D^{L}(G)=\operatorname{Tr}(G)-D(G)$ and $D^{Q}(G)=\operatorname{Tr}(G)+D(G)$, respectively. Let $\partial_{1}\geq\cdots\geq\partial_{n}$, $\partial_{1}^{L}\geq \cdots\geq\partial_{n}^{L}$ and $\partial_{1}^{Q}\geq\cdots\geq \partial_{n}^{Q}$ are the distance eigenvalues (see [1–3]), distance Laplacian eigenvalues (see [4]) and distance signless Laplacian eigenvalues (see [5]) of *G*, respectively. In particular, the eigenvalues $\partial_{1}$, $\partial_{1}^{L}$ and $\partial_{1}^{Q}$ are called the distance spectral radius, the distance Laplacian spectral radius and the distance signless Laplacian spectral radius of *G*, respectively.

Recently, remoteness, which is one of the most important distance graph parameters, has attracted much attention of many graph theory researchers. In [6], Sedlar et al. proved two AutoGraphiX (a software package devoted to conjecture-making in graph theory) conjectures on remoteness, vertex connectivity and algebraic connectivity. Sedlar [7] also studied AutoGraphiX conjectures involving remoteness and other distance invariants. Aouchiche and Hansen [8] gave Nordhaus–Gaddum-type inequalities for remoteness in graphs and the extremal graphs were also characterized. Hua et al. [9, 10] solved several conjectures related to remoteness and used remoteness to give a new sufficient condition for a connected bipartite graph to be Hamiltonian. Aouchiche and Hansen [11] provided the lower bounds on $\partial_{1}-\rho$ and $\rho+\partial_{2}$. Furthermore, they also proposed two conjectures. Lin et al. [12] confirmed these two conjectures. They also gave lower bounds on $\rho+\partial_{n}$ and $\partial_{1}-\rho$ when $G\ncong K_{n}$ and the extremal graphs were characterized. Inspired by these two papers, we continue to study the relations between remoteness and distance, distance (signless) Laplacian eigenvalues. In particular, we give lower bounds on $\rho+\partial_{1}$, $\rho-\partial_{n}$, $\rho+\partial_{1}^{L}$, $\partial_{1}^{L}-\rho$, $2\rho+\partial _{1}^{Q}$ and $\partial_{1}^{Q}-2\rho$ and the corresponding extremal graphs are characterized.

## Preliminaries

Before giving the proof of our theorems, we introduce some fundamental lemmas and properties in this section.

### Lemma 2.1

([12])

*Let* *G* *be a connected graph of order* *n* *with diameter* *d* *and remoteness ρ*. *Then*

$$\rho>\frac{d}{2}. $$

Denote by $H_{n-d}$ ($n>d$) a graph of order $n-d$ such that $V(H_{n-d})=V( \overline{K}_{n-d})$ and $E(H_{n-d})\supseteq E(\overline{K}_{n-d})$, where $\overline{K}_{n-d}$ is a null graph of order $n-d$. Let $H_{n, d}$ be a graph of order *n* with diameter *d* obtained by joining $n-d$ edges between one end of the path $P_{d}$ with each vertex of $H_{n-d}$.

### Lemma 2.2

([12])

*Let* *G* *be a connected graph of order* *n* *with diameter* *d* *and remoteness ρ*. *Then*

$$\rho\leq d-\frac{d^{2}-d}{2(n-1)} $$

*with equality holding if and only if* $G\cong H_{n, d}$.

### Lemma 2.3

([12])

*Let* *G* *be a connected graph of order* *n* *with diameter* $d\geq3$. *Then*

$$\partial_{1}>n-2+d. $$

### Lemma 2.4

([13])

*Let* *G* *be a connected graph of order* *n* *with Wiener index W*. *Then*

$$\partial_{1}\geq\frac{2W}{n}, $$

*and the equality holds if and only if* *G* *is transmission regular*.

### Lemma 2.5

([14])

*Let* $G=K_{n_{1},\ldots,n_{k}}$ *be a complete k*-*partite graph*, *where* $\sum_{i=1}^{k}n_{i}=n$ *and* $2\leq k\leq n-1$. *Then the characteristic polynomial of* $D(G)$ *is*

$$P_{D}(\lambda)=(\lambda+2)^{n-k}\Biggl[\prod _{i=1}^{k}(\lambda-n_{i}+2)- \sum _{i=1}^{k} n_{i}\prod _{j=1, j\neq i}^{k}(\lambda-n_{j}+2)\Biggr]. $$

### Lemma 2.6

([4])

*Let* *G* *be a connected graph of order* *n*. *Then* $\partial_{1}^{L} \geq n$, *with equality if and only if* $G\cong K_{n}$.

### Lemma 2.7

([15])

*Let* *G* *be a connected graph of order* *n*. *Then*

$$\partial_{1}^{L}\geq D_{1}+\frac{D_{1}}{n-1}, $$

*where* $D_{1}$ *is the maximum transmission of* *G*. *Hence*

$$\partial_{1}^{L}\geq D_{1}+1, $$

*and the equality holds if and only if* $G\cong K_{n}$.

### Lemma 2.8

([5])

*Let* $G=K_{n_{1},\ldots,n_{k}}$ *be a complete k*-*partite graph*, *where* $\sum_{i=1}^{k}n_{i}=n$ *and* $2\leq k\leq n-1$. *Then the characteristic polynomial of* $D^{L}(G)$ *is*

$$P_{D^{L}}(\mu)=\mu(\mu-n)^{k-1}\prod _{i=1}^{k}(\mu-n+n_{i})^{n _{i}-1}. $$

### Lemma 2.9

([16])

*If* *G* *is a connected graph on* $n\geq2$ *vertices*. *Then* $\partial_{1} ^{Q}\geq2n-2$ *with equality if and only if* $G\cong K_{n}$.

### Lemma 2.10

([5])

*Let* *G* *be a connected graph of order* *n* *with diameter* $d\geq3$. *Then*

$$\partial_{1}^{Q}>2n-4+2d. $$

### Lemma 2.11

([17])

*Let* *G* *be a connected graph of order* *n* *with Wiener index W*. *Then*

$$\partial_{1}^{Q}\geq\frac{4W}{n}, $$

*and the equality holds if and only if* *G* *is transmission regular*.

### Lemma 2.12

([5])

*Let* $G=K_{{n_{1},\ldots,n_{k}}}$ *be a complete k*-*partite graph*, *where* $\sum_{i=1}^{k}n_{i}=n$ *and* $2\leq k\leq n-1$. *Then the characteristic polynomial of* $D^{Q}(G)$ *is*

$$P_{D^{Q}}(q)=\prod_{i=1}^{k}(q-n-n_{i}+4)^{n_{i}-1} \Biggl[\prod_{i=1}^{k}(q-n-2n _{i}+4)- \sum_{i=1}^{k}n_{i} \prod_{j=1,j\neq i}^{k}(q-n-2n_{j}+4) \Biggr]. $$

## Remoteness and distance eigenvalues of a graph

Aouchiche et al. [11] and Lin et al. [12] obtained the following two results.

### Theorem 3.1

([11])

*Let* *G* *be a connected graph on* $n\geq4$ *vertices with the distance spectral radius* $\partial_{1}$ *and remoteness* *ρ*. *Then*

$$\partial_{1}-\rho\geq n-2 $$

*with equality if and only if* $G\cong K_{n}$.

### Theorem 3.2

([12])

*Let* *G* ($\ncong K_{n}$) *be a connected graph on* $n\geq4$ *vertices with remoteness* *ρ*. *Then*

$$\partial_{1}-\rho\geq\frac{n-1+\sqrt{(n-1)^{2}+8}}{2}- \frac{n}{n-1} $$

*with equality holding if and only if* $G\cong K_{n}-e$, *where* *e* *is an edge of* *G*.

Naturally, we consider the bounds on $\rho+\partial_{1}$ in this paper.

### Theorem 3.3

*Let* *G* *be a connected graph of order* $n\geq4$ *with remoteness* *ρ*. *Then*

$$n\leq\rho+\partial_{1}\leq\rho(P_{n})+ \partial_{1}(P_{n}) $$

*with the left equality holding if and only if* $G\cong K_{n}$ *and the right equality holding if and only if* $G\cong P_{n}$.

### Proof

By Lemma 2.1, $\rho\leq d-\frac{d^{2}-d}{2(n-1)}=f(d)$. Note that $f(d)$ is a strictly increasing function on *d*, then $\rho \leq f(n-1)=\frac{n}{2}$, and the equality holds if and only if $G\cong H_{n,n-1}=P_{n}$. Meanwhile, it was shown in [18] that $\partial_{1}(G)\leq\partial_{1}(P_{n})$ with equality holding if and only if $G\cong P_{n}$. Hence the right side of the theorem holds.

By the definition of *ρ*, we have $\rho\geq1$ with the equality if and only if $G\cong K_{n}$. As is well known [18] $\partial_{1}(G)\geq n-1$ with equality if and only if $G\cong K_{n}$. So the lower bound is completed. □

### Theorem 3.4

*Let* *G* ($\ncong K_{n}$) *be a connected graph of order* $n\geq4$ *with remoteness* *ρ*. *Then*

$$\rho+\partial_{1}\geq\frac{n}{n-1}+ \frac{n-1+\sqrt{(n-1)^{2}+8}}{2} $$

*with equality holding if and only if* $G\cong K_{n}-e$.

### Proof

For $G\cong K_{n}-e$, note that $K_{n}-e$ is a complete multipartite graph, by Lemma 2.5, then

$$\rho+\partial_{1}=\frac{n}{n-1}+\frac{n-1+\sqrt{(n-1)^{2}+8}}{2}< 1+ \frac{1}{n-1}+\frac{2(n-1)+\frac{4}{n-1}}{2}=n+\frac{3}{n-1}. $$

Let *G* be a connected graph with diameter *d*. If $d\geq3$, by Lemmas 2.1 and 2.3, then

$$\rho+\partial_{1}>\frac{d}{2}+n-2+d=\frac{3}{2}d+n-2>n+ \frac{3}{n-1}> \rho(K_{n}-e)+\partial_{1}(K_{n}-e). $$

If $d=2$, we have

$$\rho=\frac{\delta+2(n-1-\delta)}{n-1}=\frac{2(n-1)-\delta }{n-1}=2-\frac{ \delta}{n-1}. $$

By Lemma 2.4, then

$$\partial_{1}\geq\frac{2W}{n}\geq\frac{n(n-1)+2(n-1-\delta)}{n}=n+1- \frac{2(1+ \delta)}{n}. $$

Hence

$$\rho+\partial_{1}\geq n+3-\frac{2(1+\delta)}{n}-\frac{\delta}{n-1}. $$

If $\delta\leq n-3$, then

$$\rho+\partial_{1}\geq n+3-\frac{2(n-2)}{n}-\frac{n-3}{n-1}=n+ \frac{4}{n}+\frac{2}{n-1}>n+\frac{3}{n-1}>\rho(K_{n}-e)+ \partial_{1}(K _{n}-e). $$

If $\delta=n-2$, we have $\rho(G)=\rho(K_{n}-e)$ and $\partial_{1}(G)> \partial_{1}(G+e)$, and thus $\rho(G)+\partial_{1}(G)\geq\rho(K _{n}-e)+\partial_{1}(K_{n}-e)$ with equality if and only if $G\cong K_{n}-e$. The result follows. □

### Theorem 3.5

*Let* *G* ($\ncong K_{n},K_{n}-e$) *be a connected graph of order* $n\geq4$ *with remoteness* *ρ*. *Then*

$$\rho+\partial_{1}\geq\frac{n}{n-1}+ \frac{n-1+\sqrt{(n-1)^{2}+16}}{2} $$

*with equality holding if and only if* $G\cong K_{n}-2e$, *where* 2*e* *are two matching edges*.

### Proof

For $G\cong K_{n}-2e$,

$$\rho+\partial_{1}=\frac{n}{n-1}+\frac{n-1+\sqrt{(n-1)^{2}+16}}{2}< \frac{n}{n-1}+\frac{2(n-1)+\frac{8}{n-1}}{2}=n+\frac{5}{n-1}. $$

Let *G* be a connected graph with *d*. If $d\geq3$, then

$$\rho+\partial_{1}>\frac{d}{2}+n-2+d=\frac{3}{2}d+n-2>n+ \frac{5}{n-1}> \rho(K_{n}-2e)+\partial_{1}(K_{n}-2e). $$

If $d=2$, similarly we have

$$\rho+\partial_{1}\geq n+3-\frac{2(1+\delta)}{n}-\frac{\delta}{n-1}. $$

If $\delta\leq n-3$, then

$$\rho+\partial_{1}\geq n+3-\frac{2(n-2)}{n}-\frac{n-3}{n-1}=n+ \frac{4}{n}+\frac{2}{n-1}\geq n+\frac{5}{n-1}>\rho (K_{n}-2e)+\partial _{1}(K_{n}-2e). $$

If $\delta=n-2$, then $\partial_{1}(G)>\partial_{1}(G+e)$ and $\rho(G)=\rho(K_{n}-2e)$, and hence $\partial_{1}(G)+\rho(G)\geq \partial_{1}(K_{n}-2e)+\rho(K_{n}-2e)$ with equality holding if and only if $G\cong K_{n}-2e$. □

In [11], Aouchiche and Hansen showed a lower bound on the sum of the remoteness and the second largest distance eigenvalue, $\rho+\partial_{2}$, of a graph with given number of vertices *n*.

### Theorem 3.6

([11])

*Let* *G* *be a connected graph of order* $n\geq4$ *with remoteness* *ρ*. *Then*

$$\rho+\partial_{2}\geq0, $$

*with equality holding if and only if* $G\cong K_{n}$.

In fact, the bound in the above corollary is best possible among the bounds of the form $\rho+\partial_{k}\geq0$, with a fixed integer *k*, over the class of all connected graphs. First, we prove a lower bound on $\rho+\partial_{2}$ among all the complete bipartite graphs $K_{a,b}$.

### Theorem 3.7

*Let* *G* *be a complete bipartite graph of order* $n\geq4$ *with remoteness* *ρ*. *Then*

$$\rho(K_{a,b})+\partial_{2}(K_{a,b})\geq n- \frac{1}{n-1}-\sqrt{n ^{2}-3n+3} $$

*with equality holding if and only if* $G\cong K_{1,n-1}$.

### Proof

For $G\cong K_{a,b}$, where $1\leq a\leq\lfloor\frac{n}{2}\rfloor$, we have

$$\rho(K_{a,b})+\partial_{2}(K_{a,b})=n- \frac{a}{n-1}-\sqrt{3a^{2}-3na+n ^{2}}. $$

Let $f(a)=n-\frac{a}{n-1}-\sqrt{3a^{2}-3na+n^{2}}$. By a direct calculation, $f''(a)<0$, $f'(0)>0$ and $f'(\frac{n}{2})<0$. Then there exists a zero root $a_{0}$ such that $f'(a_{0})=0$. So $f(a)$ is an increasing function of *a* on $[0, a_{0}]$ and is a decreasing function on $[a_{0}, \lfloor\frac{n}{2}\rfloor]$. Note that $1< a_{0}$ and $f(1)< f(\lfloor\frac{n}{2}\rfloor)$. Hence $\rho(K_{a,b})+\partial _{2}(K_{a,b})\geq f(1)=n-\frac{1}{n-1}-\sqrt{n^{2}-3n+3}$, with equality holding if and only if $G\cong K_{1,n-1}$. □

Naturally, for a connected graph, we propose the following conjecture.

### Conjecture 3.8

*Let* *G* ($\ncong K_{n},K_{n}-e$) *be a connected graph of order* $n\geq4$ *with remoteness ρ*. *Then*

$$\rho+\partial_{2}\geq\frac{n}{n-1}+ \frac{n-1-\sqrt{(n-1)^{2}+8}}{2}, $$

*with equality holding if and only if* $G\cong K_{n}-2e$, *where* 2*e* *are two matching edges*.

Furthermore, Aouchiche [11] et al. proved the following result.

### Theorem 3.9

([11])

*Let* *G* *be a connected graph on* $n\geq4$ *vertices with the least distance eigenvalues* $\partial_{n}$ *and remoteness* *ρ*. *Then*

$$\rho+\partial_{n}\leq0 $$

*with equality if and only if* $G\cong K_{n}$.

Lin [19] showed that $\partial_{n}\leq-d$ with equality if and only if *G* is a complete multipartite graph. Using this, we obtain the following result.

### Theorem 3.10

*Let* *G* ($\ncong K_{n}$) *be a connected graph of order* $n\geq4$ *with remoteness* *ρ*. *Then*

$$\rho+\partial_{n}\leq-\frac{1}{n-1} $$

*with equality holding if and only if* $G\cong K_{1,n-1}$.

### Proof

Let *G* be a connected graph with diameter *d*. Then $2\leq d\leq n-1$. Clearly, by Lemma 2.2, we know that $\rho+\partial_{n}\leq -\frac{d ^{2}-d}{2(n-1)}=f(d)$. Note that $f(d)$ is a strictly decreasing function on *d*. Hence $\rho+\partial_{n}\leq- \frac{d^{2}-d}{2(n-1)}\leq f(2)=-\frac{1}{n-1}$, with the equality holding if and only if $G\cong H_{n,2}$ and *G* is a complete multipartite graph. Thus $G\cong K_{1,n-1}$. □

Next, we start to consider the lower bound on $\rho-\partial_{n}$.

### Theorem 3.11

*Let* *G* *be a connected graph of order* $n\geq4$ *with remoteness* *ρ*. *Then*

$$\rho-\partial_{n}\geq2 $$

*with equality holding if and only if* $G\cong K_{n}$.

### Proof

Let *G* be a connected graph with *d*. If $d\geq2$, then $\rho- \partial_{n}>\frac{d}{2}+d=\frac{3}{2}d\geq3$. Note that $\rho(K _{n})-\partial_{n}(K_{n})=2$, the result follows. □

### Theorem 3.12

*Let* *G* ($\ncong K_{n}$) *be a connected graph of order* $n\geq4$ *with remoteness* *ρ*. *Then*

$$\rho-\partial_{n}\geq3+\frac{1}{n-1} $$

*with equality holding if and only if* $G\cong K_{n}-me$, *where* *me* *denotes* *m* *matching edges*.

### Proof

Note that $\rho(K_{n}-me)-\partial_{n}(K_{n}-me)=\frac{n}{n-1}+2=3+ \frac{1}{n-1}$. Let *G* be a connected graph with *d*. If $d\geq3$, then

$$\rho-\partial_{n}>\frac{d}{2}+d=\frac{3}{2}d\geq \frac{9}{2}>3+ \frac{1}{n-1}. $$

If $d=2$, then $\rho=2-\frac{\delta}{n-1}$, and hence $\rho- \partial_{n}\geq2-\frac{\delta}{n-1}+d=4-\frac{\delta}{n-1}$. If $\delta\leq n-3$, we have $\rho-\partial_{n}>3+\frac{1}{n-1}$. If $\delta=n-2$, then $G\cong K_{n}-me$, where *me* are *m* matching edges. This completes the proof. □

## Remoteness and distance Laplacian eigenvalues of a graph

In this section, we mainly investigate the relations between remoteness and the distance Laplacian eigenvalues of a graph.

### Theorem 4.1

*Let* *G* *be a connected graph of order* $n\geq4$ *with remoteness* *ρ*. *Then*

$$n+1\leq\rho+\partial_{1}^{L}\leq\rho(P_{n})+ \partial_{1}^{L}(P _{n}) $$

*with the left equality holding if and only if* $G\cong K_{n}$ *and the right equality holding if and only if* $G\cong P_{n}$.

### Proof

By Lemma 2.6, we have $\rho+\partial_{1}^{L}\geq\rho+n \geq n+1$, with the left equality holding if and only if $G\cong K _{n}$.

Similar to the proof of Theorem 3.3, we have $\rho(G)\leq \rho(P_{n})$ with the equality if and only if $G\cong P_{n}$. Meanwhile, it was shown in [20] that $\partial_{1}^{L}(G) \leq\partial_{1}^{L}(P_{n})$ with equality holding if and only if $G\cong P_{n}$. Hence the right side of the theorem holds. □

### Theorem 4.2

*Let* *G* ($\ncong K_{n}$) *be a connected graph of order* $n\geq4$ *with remoteness* *ρ*. *Then*

$$\rho+\partial_{1}^{L}\geq n+\frac{1}{n-1}+3 $$

*with equality holding if and only if* $G\cong K_{n}-me$, *where* *me* *denotes* *m* *matching edges*.

### Proof

For $G\cong K_{n}-me$, note that $K_{n}-me$ is a complete multipartite graph, by Lemma 2.8, then $\rho+\partial_{1}^{L}= \frac{n}{n-1}+n+2=n+\frac{1}{n-1}+3$. Let *G* be a connected graph with *d*. If $d\geq3$, by Lemma 2.7, we obtain

$$\rho+\partial_{1}^{L}\geq\rho+D_{1}+ \frac{D_{1}}{n-1}=(n+1)\rho> \frac{d}{2}(n+1)\geq\frac{3}{2}(n+1)>n+ \frac{1}{n-1}+3. $$

If $d=2$, then $\rho+\partial_{1}^{L}\geq(n+1)\rho=(n+1)(2-\frac{ \delta}{n-1})$. If $\delta\leq n-3$, then

$$\rho+\partial_{1}^{L}\geq(n+1) \biggl(2-\frac{n-3}{n-1} \biggr)=n+\frac{4}{n-1}+3>n+ \frac{1}{n-1}+3. $$

If $\delta=n-2$, then $G\cong K_{n}-me$. The result follows. □

### Theorem 4.3

*Let* *G* *be a connected graph of order* $n\geq4$ *with remoteness* *ρ*. *Then*

$$\partial_{1}^{L}-\rho\geq n-1 $$

*with equality holding if and only if* $G\cong K_{n}$.

### Proof

By Lemma 2.7, we have

$$\partial_{1}^{L}-\rho\geq D_{1}+1-\rho=(n-2) \rho+1\geq n-1, $$

with equality holding if and only if $G\cong K_{n}$. □

### Theorem 4.4

*Let* *G* ($\ncong K_{n}$) *be a connected graph of order* $n\geq5$ *with remoteness* *ρ*. *Then*

$$\partial_{1}^{L}-\rho\geq n+1-\frac{1}{n-1} $$

*with equality if and only if* $G\cong K_{n}-me$, *where* $1\leq m\leq \lfloor\frac{n}{2}\rfloor$.

### Proof

If $G\cong K_{n}-me$, then $\partial_{1}^{L}-\rho=n+2-\frac{n}{n-1}=n+1- \frac{1}{n-1}$. Let *G* be a connected graph with *d*. If $d\geq3$, then

$$\partial_{1}^{L}-\rho\geq D_{1}+ \frac{D_{1}}{n-1}-\rho=(n-1)\rho> \frac{3}{2}(n-1)>n+1-\frac{1}{n-1}. $$

If $d=2$, then $\partial_{1}^{L}-\rho\geq(n-1)\rho=(n-1)(2-\frac{ \delta}{n-1})$. If $\delta\leq n-3$, then $\partial_{1}^{L}-\rho \geq(n-1)(2-\frac{n-3}{n-1})=n+1>n+1-\frac{1}{n-1}$. If $\delta=n-2$, then $G\cong K_{n}-me$. This completes the proof. □

## Remoteness and distance signless Laplacian eigenvalues of a graph

First, we consider the bounds on $2\rho+\partial _{1}^{Q}$.

### Theorem 5.1

*Let* *G* *be a connected graph of order* $n\geq4$ *with remoteness* *ρ*. *Then*

$$2n\leq2\rho+\partial_{1}^{Q}\leq2\rho(P_{n})+ \partial_{1}^{Q}(P _{n}) $$

*with the left equality holding if and only if* $G\cong K_{n}$ *and the right equality holding if and only if* $G\cong P_{n}$.

### Proof

For $G\cong K_{n}$, then $2\rho+\partial_{1}^{Q}=2n-2+2=2n$.

If $d\geq3$, we know that $\partial_{1}^{Q}(G)>2n-4+2d\geq2n+2$, thus $\partial_{1}^{Q}(G)+2\rho>2n$.

If $d=2$, we know that $\rho=2-\frac{\delta}{n-1}$ and $\partial _{1}^{Q}\geq\frac{4W}{n}\geq2(n+1)-\frac{4(1+\delta)}{n}$. Then $\partial_{1}^{Q}(G)+2\rho=2n+2-\frac{4(1+\delta)}{n}+4-\frac{2 \delta}{n-1}=2n+6-\frac{4(1+\delta)}{n}-\frac{2\delta}{n-1}>2n$.

It was shown in [20] that $\partial_{1}^{Q}(G)\leq\partial _{1}^{Q}(P_{n})$ with equality holding if and only if $G\cong P_{n}$. Hence the right side of the theorem holds. □

### Theorem 5.2

*Let* *G* ($\ncong K_{n}$) *be a connected graph of order* $n\geq4$ *with remoteness* *ρ*. *Then*

$$2\rho+\partial_{1}^{Q}\geq\frac{3n-2+\sqrt{(n-2)^{2}+16}}{2}+ \frac{2n}{n-1} $$

*with equality holding if and only if* $G\cong K_{n}-e$.

### Proof

For $G\cong K_{n}-e$, by Lemma 2.12, then

$$\begin{aligned} 2\rho+\partial_{1}^{Q} =&\frac{3n-2+\sqrt{(n-2)^{2}+16}}{2}+ \frac{2n}{n-1}< \frac{3n-2+n-2+\frac{8}{n-2}}{2}+\frac{2n}{n-1} \\ =&2n+\frac{4}{n-2}+\frac{2}{n-1}. \end{aligned}$$

Let *G* be a connected graph with *d*. If $d\geq3$, by Lemma 2.10, then

$$2\rho+\partial_{1}^{Q}>2n-4+2d+d\geq2n+5>2n+ \frac{4}{n-2}+ \frac{2}{n-1}. $$

If $d=2$, then $\rho=2-\frac{\delta}{n-1}$. By Lemma 2.11, $\partial_{1}^{Q}\geq\frac{4W}{n}\geq2n+2-\frac{4(1+\delta)}{n}$. Thus

$$2\rho+\partial_{1}^{Q}\geq2n+2-\frac{4(1+\delta)}{n}+4- \frac{2 \delta}{n-1}=2n+6-\frac{4(1+\delta)}{n}-\frac{2\delta}{n-1}. $$

If $\delta\leq n-3$, then $2\rho+\partial_{1}^{Q}\geq2n+6- \frac{4(n-2)}{n}-\frac{2(n-3)}{n-1}=2n+\frac{8}{n}+\frac{4}{n-1}>2n+ \frac{4}{n-2}+\frac{2}{n-1}$. If $\delta=n-2$, we know that $\rho(G)=\rho(K_{n}-e)$ and $\partial_{1}^{Q}(G)>\partial_{1}^{Q}(G+e)$, thus $2\rho(G)+\partial _{1}^{Q}(G)\geq2\rho(K_{n}-e)+\partial_{1}^{Q}(K_{n}-e)$ with equality if and only if $G\cong K_{n}-e$. □

### Theorem 5.3

*Let* *G* ($\ncong K_{n},K_{n}-e$) *be a connected graph of order* $n\geq4$ *with remoteness* *ρ*. *Then*

$$2\rho+\partial_{1}^{Q}\geq\frac{3n-2+\sqrt{(n-2)^{2}+32}}{2}+ \frac{2n}{n-1} $$

*with equality holding if and only if* $G\cong K_{n}-2e$, *where* 2*e* *are two matching edges*.

### Proof

For $G\cong K_{n}-2e$, then

$$\begin{aligned} 2\rho+\partial_{1}^{Q} =&\frac{3n-2+\sqrt{(n-2)^{2}+32}}{2}+ \frac{2n}{n-1} \\ < &\frac{3n-2+n-2+\frac{16}{n-2}}{2}+\frac{2n}{n-1} \\ =&2n+\frac{8}{n-2}+\frac{2}{n-1}. \end{aligned}$$

Let *G* be a connected graph with *d*. If $d\geq3$, then

$$2\rho+\partial_{1}^{Q}(G)>2n-4+2d+d\geq2n+5>2n+ \frac{8}{n-2}+ \frac{2}{n-1}. $$

If $d=2$, then $\rho=2-\frac{\delta}{n-1}$, $\partial_{1}^{Q}(G) \geq2n+2-\frac{4(1+\delta)}{n}$, and thus

$$\partial_{1}^{Q}(G)+2\rho\geq2n+2-\frac{4(1+\delta)}{n}+4- \frac{2 \delta}{n-1}=2n+6-\frac{4(1+\delta)}{n}-\frac{2\delta}{n-1}. $$

If $\delta\leq n-3$, then $\partial_{1}^{Q}(G)+2\rho\geq2n+6- \frac{4(n-2)}{n}-\frac{2(n-3)}{n-1}=2n+\frac{8}{n}+\frac{4}{n-1}>2n+ \frac{8}{n-2}+\frac{2}{n-1}$. If $\delta=n-2$, we have $\rho(G)= \rho(K_{n}-2e)$ and $\partial_{1}^{Q}(G)>\partial_{1}^{Q}(G+e)$, thus $\partial_{1}^{Q}(G)+2\rho(G)\geq\partial_{1}^{Q}(K_{n}-2e)+2\rho(K _{n}-2e)$ with equality holding if and only if $G\cong K_{n}-2e$. This completes the proof. □

Next, we prove the lower bound on $\partial_{1}^{Q}-2\rho$.

### Theorem 5.4

*Let* *G* *be a connected graph of order* $n\geq4$ *with remoteness* *ρ*. *Then*

$$\partial_{1}^{Q}-2\rho\geq2n-4 $$

*with equality holding if and only if* $G\cong K_{n}$.

### Proof

By Lemma 2.11, we have

$$\begin{aligned} \partial_{1}^{Q} \geq&\frac{4W(G)}{n}= \frac{2\sum_{v\in V(G)}\operatorname{Tr}(v)}{n}=\frac{2(2(n-1)\rho+\sum_{2\leq i,j\leq n}d_{ij})}{n} \\ \geq&\frac{4(n-1)\rho+2(n-1)(n-2)}{n} \\ =& \frac{2n\rho+2(n-2)\rho+2n(n-2)-2(n-2)}{n} \\ =&2\rho+2(n-2)+\frac{2(n-2)(\rho-1)}{n} \geq2\rho+2n-4. \end{aligned}$$

Thus $\partial_{1}^{Q}-2\rho\geq2n-4$ with equality if and only if $G\cong K_{n}$. □

### Theorem 5.5

*Let* *G* ($\ncong K_{n}$) *be a connected graph of order* $n\geq4$ *with remoteness* *ρ*. *Then*

$$\partial_{1}^{Q}-2\rho\geq\frac{3n-2+\sqrt{(n-2)^{2}+16}}{2}- \frac{2n}{n-1} $$

*with equality holding if and only if* $G\cong K_{n}-e$.

### Proof

For $G\cong K_{n}-e$, then we have

$$\partial_{1}^{Q}-2\rho=\frac{3n-2+\sqrt{(n-2)^{2}+16}}{2}- \frac{2n}{n-1}< 2n-4+\frac{4}{n-2}-\frac{2}{n-1}. $$

Let *G* be a connected graph with *d*. If $d\geq3$, by Lemmas 2.2 and 2.10, then

$$\partial_{1}^{Q}-2\rho>2n-4+2d-2d+\frac{d^{2}-d}{n-1} \geq2n-4+ \frac{6}{n-1}>2n-4+\frac{4}{n-2}-\frac{2}{n-1}. $$

If $d=2$, then

$$\partial_{1}^{Q}-2\rho\geq2n-2-\frac{4(1+\delta)}{n}+ \frac {2\delta }{n-1}=2n-2-\frac{4}{n}-\biggl(\frac{4}{n}- \frac{2}{n-1}\biggr)\delta. $$

If $\delta\leq n-3$, then

$$\partial_{1}^{Q}-2\rho\geq2n-2-\frac{4}{n}-\biggl( \frac{4}{n}- \frac{2}{n-1}\biggr) (n-3)>2n-4+\frac{4}{n-2}- \frac{2}{n-1}. $$

If $\delta=n-2$, we know that $\rho(G)=\rho(K_{n}-e)$ and $\partial_{1}^{Q}(G)>\partial_{1}^{Q}(G+e)$, thus $\partial_{1}^{Q}(G)-2 \rho(G)\geq\partial_{1}^{Q}(K_{n}-e)-2\rho(K_{n}-e)$ with equality holding if and only if $G\cong K_{n}-e$. This completes the proof. □

## Conclusions

We give lower bounds on $\rho+\partial_{1}$, $\rho-\partial_{n}$, $\rho+\partial_{1}^{L}$, $\partial_{1}^{L}-\rho$, $2\rho+\partial _{1}^{Q}$ and $\partial_{1}^{Q}-2\rho$ and the corresponding extremal graphs are characterized. Considering the distance, distance Laplacian and distance signless Laplacian eigenvalues of a graph is still an interesting and important problem.
